# A Novel Mobile App for Personalized Dietary Advice Leveraging Persuasive Technology, Computer Vision, and Cloud Computing: Development and Usability Study

**DOI:** 10.2196/46839

**Published:** 2023-08-07

**Authors:** Vivienne Guan, Chenghuai Zhou, Hengyi Wan, Rengui Zhou, Dongfa Zhang, Sihan Zhang, Wangli Yang, Bhanu Prakash Voutharoja, Lei Wang, Khin Than Win, Peng Wang

**Affiliations:** 1 School of Medical, Indigenous and Health Sciences Faculty of Science, Medicine and Health University of Wollongong Wollongong, New South Wales Australia; 2 School of Computing and Information Technology Faculty of Engineering and Information Sciences University of Wollongong Wollongong, New South Wales Australia

**Keywords:** food, diet, mobile health, mHealth, persuasive technology, gamification, computer vision, cloud computing, design science research, mobile phone

## Abstract

**Background:**

The Australian Dietary Guidelines (ADG) translate the best available evidence in nutrition into food choice recommendations. However, adherence to the ADG is poor in Australia. Given that following a healthy diet can be a potentially cost-effective strategy for lowering the risk of chronic diseases, there is an urgent need to develop novel technologies for individuals to improve their adherence to the ADG.

**Objective:**

This study describes the development process and design of a prototype mobile app for personalized dietary advice based on the ADG for adults in Australia, with the aim of exploring the usability of the prototype. The goal of the prototype was to provide personalized, evidence-based support for self-managing food choices in real time.

**Methods:**

The guidelines of the design science paradigm were applied to guide the design, development, and evaluation of a progressive web app using Amazon Web Services Elastic Compute Cloud services via iterations. The food layer of the Nutrition Care Process, the strategies of cognitive behavioral theory, and the ADG were translated into prototype features guided by the Persuasive Systems Design model. A gain-framed approach was adopted to promote positive behavior changes. A cross-modal image-to-recipe retrieval model under an Apache 2.0 license was deployed for dietary assessment. A survey using the Mobile Application Rating Scale and semistructured in-depth interviews were conducted to explore the usability of the prototype through convenience sampling (N=15).

**Results:**

The prominent features of the prototype included the use of image-based dietary assessment, food choice tracking with immediate feedback leveraging gamification principles, personal goal setting for food choices, and the provision of recipe ideas and information on the ADG. The overall prototype quality score was “acceptable,” with a median of 3.46 (IQR 2.78-3.81) out of 5 points. The median score of the perceived impact of the prototype on healthy eating based on the ADG was 3.83 (IQR 2.75-4.08) out of 5 points. In-depth interviews identified the use of gamification for tracking food choices and innovation in the image-based dietary assessment as the main drivers of the positive user experience of using the prototype.

**Conclusions:**

A novel evidence-based prototype mobile app was successfully developed by leveraging a cross-disciplinary collaboration. A detailed description of the development process and design of the prototype enhances its transparency and provides detailed insights into its creation. This study provides a valuable example of the development of a novel, evidence-based app for personalized dietary advice on food choices using recent advancements in computer vision. A revised version of this prototype is currently under development.

## Introduction

### Background

In 2017, approximately 11 million deaths and 255 million disability-adjusted life years were attributable to dietary risk factors worldwide [1]. Poor food intake contributes to 5 of the top 6 chronic disease risk factors (eg, obesity and high blood pressure) in Australia [2]. This poor intake is accountable for over 40% of the heart disease and 34% of the endocrine disease burden in Australia [2]. Healthy eating behaviors, such as increased fruit and vegetable consumption, are associated with a reduced risk of cardiovascular diseases and certain cancers [2].

National dietary guidelines such as the Australian Dietary Guidelines (ADG) are intended to influence the dietary behaviors of the general population to maintain health and inform food, nutrition, and health policies and programs [3]. The ADG are statements to translate the best available evidence for dietary patterns, foods, and nutrients for health into food choice recommendations [3]. However, adherence to the ADG is poor in Australia. For example, <10% of Australian adults met the recommendations for daily vegetable consumption in 2020-2021, and this low rate has remained consistent over time [4,5]. There are many barriers to adherence to the ADG. The current food supply is complex, with many foods available to the public. Cognitive skills, such as planning and problem-solving, are required to incorporate the dietary guidelines into the everyday practicalities associated with navigating the food system [6]. Moreover, particular circumstances are also common in today’s lifestyles, for example, being rushed, having too little sleep, and experiencing psychosocial stress [7]. These circumstances can increasingly challenge people to make healthy food choices [7]. It implies that a supportive tool is required to provide real-time personalized, practical advice for food choices, facilitating adherence to the ADG.

Mobile health (mHealth) refers to the use of mobile devices such as smartphones and tablets in health and health service delivery [8]. Mobile apps are increasingly being used to deliver and support nutrition interventions in community settings [9]. With just over 6 billion smartphone users worldwide [10], the increasing popularity of smartphones may offer new opportunities to implement innovative, scalable tools to improve the reach and effectiveness of nutrition interventions and make positive behavior changes [11-14]. The use of mobile apps for nutrition interventions has many potential advantages because of their ubiquity, cost-effectiveness, less-invasive nature, ability to provide immediate feedback, and tracking capabilities. Good adherence and usability of apps for nutrition interventions were established in controlled environments (eg, testing a mobile app); however, adherence to the intervention in real-world settings was low [15,16].

Adherence to digital interventions appears to rely heavily on the interactive design of digital tools [17,18]. Interactive apps focus on human-app interaction to support users in communicating and interacting with digital tools on a daily basis [19]. The concept of user experience has been centered on interaction design [20]. The emotional elements of interaction design tend to elicit a desirable user experience (eg, enjoyment) and avoid undesirable user experiences (eg, frustration) [19]. For example, users tend to continue using an app if it provides an enjoyable user experience, whereas frustration with an app often leads to users stopping its use. These emotional aspects of interaction can also be applied in designing an app to persuade people for behavior changes [19,21].

Persuasive technology is defined as an interactive information system designed to change people’s attitudes and behaviors with a focus on the implementation side related to the features of information systems [21]. For example, a mobile app for persuasive technology can be used as a tool to persuade users for behavior changes by making target behavior easier to perform [21]. Such an app provides a medium for gaining experience in planned interventions for behavior changes [21]. This experience assists in persuading users to motivate and rehearse a target behavior. The Persuasive Systems Design (PSD) model offers a framework for developing and evaluating persuasive systems at the interface level [22]. The PSD model categorizes the design principles of a persuasive system into primary task support (carrying out the user’s primary task), dialogue support (providing feedback and implementing computer-human dialogue support in a manner that helps users keep moving toward their goal or target behavior), social support (motivating users by leveraging social influence), and credibility support (being more credible and thus more persuasive) [22]. The aim of applying the design principles of the PSD model at the interface level is to guide the planned persuasive effects when using the tool, which facilitates communication of the nature, purpose, procedures, potential risks, and benefits of using the tool [21,22]. Furthermore, gamification refers to the use of game-like elements, such as rewards and competition, in nongame contexts to motivate individuals to engage in target behavior [23]. For example, a gamified nutrition intervention provides rewards for reaching the goals for increasing vegetable intake [24-26]. Thus, gamification can be viewed as a subfield of persuasive technology [21,22,27]. It often involves the use of technology to design and implement game-like features, with the goal of persuading and influencing users.

To evoke a desirable user experience when using an app, a user-centered design principle tends to be applied to create solutions specific to the intended users [28]. However, previous evidence has demonstrated that the apps did not meet the needs of users, although users were involved in the early design process [29]. It has been suggested that there is a gap in translating users’ specific needs for apps into technological features [30-32]. A certain level of digital literacy, including the competencies and skills required to navigate mobile devices, is required to use and interpret digital health tools [33]. For example, users may need to understand the language, hardware, and software to effectively navigate the technology and communicate their needs. Therefore, engaging users in the design process can have advantages for managing expectations and fostering a sense of ownership over an app. However, determining the appropriate timing and manner in which users are involved in the development process may require careful planning [19].

The use of behavior change theory in nutrition interventions is pivotal to the effectiveness of mHealth interventions. Commercial apps are typically service-oriented and contain an appealing user interface, but evidence reveals that they tend to have limited basis in evidence and behavior change theory [34,35]. The underlying philosophy of validated behavior change theories within the field of nutrition and dietetics offers the assumptions for behavior changes [36]. These assumptions can assist in understanding external and internal issues and the dynamics that facilitate behavior changes [36]. Specific behavior change strategies for nutrition interventions can be selected based on these insights [36]. For example, cognitive behavioral theory (CBT) [37] is one of the oldest and most tested behavior change theories used in nutrition and dietetics [36]. The literature suggests that CBT-based interventions are effective in changing targeted dietary habits, such as increasing the intake of fruits and vegetables [36]. The assumption underlying CBT is that behavior can be learned through or facilitated by cognitive and behavioral strategies [37]. Applying CBT, people tend to be taught a variety of strategies to make them aware of internal and external cues and their response [37]. Examples of strategies endorsed by CBT include self-monitoring, goal setting, problem-solving, and rewards [37]. Thus, the underlying philosophy and behavior change strategies of CBT offer a rationale for the cognitive aspects of the interaction design for an app.

Moreover, there has been growing awareness that a one-size-fits-all approach to dietary advice has limited effectiveness, and there is a need to adopt a personalized approach. In nutrition science, personalized nutrition has gained popularity in recent years. Personalized nutrition can be based on biological evidence of differential responses to food or nutrients (eg, genotypic or phenotypic characteristics) [38]. It can also be based on an analysis of current behavior, preferences, barriers, and goals related to dietary intake [38]. This personalization approach attempts to deliver a nutrition intervention at an individual level [38]. However, evidence suggests that the biological characteristics of individuals tend to act only as catalysts to encourage changes in eating behavior [38]. Eating foods is repeated multiple times during a day as meals (eg, breakfast, lunch, dinner, or snacks), which are distinguished from one another by specific food choices [39-41]. It indicates that there is a need to focus on developing supportive approaches to effectively motivate and enable users to make positive dietary changes based on an individual’s dietary intake. Despite this, the review of highly ranked 80 dietary tracking apps from the Google Play Store, Apple App Store, and Microsoft Store in 2022 demonstrated that the absence of personalized dietary advice that considers users’ dietary intake is a notable gap in the features of these apps [42].

The limitations of the available apps for nutrition interventions indicate that obtaining high-quality dietary intake data is central to the design of an app for personalized dietary advice. In the food layer of the Nutrition Care Process (NCP), Nutrition Assessment is the first step to systematically collect timely and relevant dietary intake data needed to identify nutrition-related problems and causes (Nutrition Diagnosis) [43]. However, diet is complex because of the different foods that are consumed in varying portions, fluctuating quantities over time, and differing combinations [39,44-46]. There is a trade-off between user burdens and the level of details required for collecting dietary intake data because tracking of dietary intake usually requires substantial involvement from users [44,45]. Furthermore, reliable food composition data are required to assess dietary intake, which is the key determinant of the quality of apps [35].

The widespread use of smartphones with integrated high-quality cameras has driven the inclusion of technology-assisted dietary assessments in nutrition apps. The use of these methods reduces user burden and maximizes the effectiveness of tracking dietary intake over time [47]. The review of highly ranked 80 dietary tracking apps from the app stores in 2022 demonstrated that 51% of apps have a barcode scanner for food recognition [42]. However, barcode scanning is not suitable for all types of food (eg, mixed dishes and unpacked foods) and cannot provide the actual portion of consumed food. Despite the significant advancements in using computer vision to automatically assess dietary intake from food photos [48], their application beyond academic settings is still quite limited. For instance, 3 out of the reviewed 80 commercial apps in 2022 use this technique, with only 1 app, Foodvisor, featuring automatic recognition and nutritional information calculation from food photos [42].

In recent years, image recognition (eg, food items and portion size estimation) has undergone a paradigm shift toward using deep learning for its strong capability in feature learning of images to achieve greater performance [48,49]. However, food recognition from food images is still unable to achieve optimal performance, particularly for mixed foods such as foods containing more than 1 ingredient [48,49]. Food recognition from food images requires fine-grained ingredient recognition. Directly recognizing ingredients is challenging, because ingredients from prepared food are mixed during food preparation. Furthermore, cutting and cooking food may change the surface color, shape, and texture of food. Using image-to-recipe retrieval, computer vision techniques analyze the visual characteristics of food images and establish a connection between the rich contextual details found in recipes with the visual content presented in food images [48]. A larger data set with full recipe information extracted from the internet was used in a study of image-to-recipe retrieval to identify multiple foods and ingredients in a food image with 93% accuracy [50].

### Objectives

This study describes the development process and design of a prototype mobile app for personalized dietary advice based on the ADG for adults in Australia, with the aim of exploring the usability of the prototype. The goal of the prototype was to provide personalized evidence-based support for self-managing food choices in real time.

## Methods

### Overview

The design science paradigm incorporates behavioral aspects into the design of information systems to solve real-world problems [51]. This approach is well suited for designing, developing, and evaluating mobile apps because it provides a conceptual framework, along with flexible guidelines for understanding, executing, and evaluating an information system [51]. Thus, the guidelines of the design science paradigm were applied to guide the design, development, and evaluation of the prototype in 3 phases, including the determination of the requirements (Guideline 2: Problem relevance and Guideline 5: Research rigor), development of the prototype (Guideline 6: Design as a search process and Guideline 1: Design as an artifact), and evaluation of the prototype (Guideline 3: Design evaluation). A co-design approach was applied by a team of researchers in computer science and information technology as well as an Accredited Practising Dietitian (APD), with the latter taking the lead.

### Determination of Prototype Requirements

The lead dietitian (VG) developed the initial requirements of the prototype based on her previous work on food choices and the flow of generating dietary intake data using detailed dietary intake data, gold standard biomarkers, mixed research methods, and a data-mining tool [39,44-46,52]. The requirements of the prototype were shaped by the integration of the food layer of the NCP [43], the strategies of CBT [37], and the ADG [3], along with insights from the reviews of the characteristics of apps for nutrition interventions [9,47]. A gain-framed approach was adopted to guide the design of the prototype, with the aim of promoting positive behavior changes (eg, increase in awareness and motivation for healthy eating, as well as improve self-management skills and nutrition knowledge) and avoiding negative outcomes (eg, obsessive energy counting and feelings of guilt or disappointment) [53]. The PSD model [22] was used to guide the interaction design at the interface level. The final requirements of the app were defined by the research team.

The wireframes of each screen were developed. The wireframes focused on the screen layout and were organized to reflect the food layer of the NCP. The wireframes were then mapped to mock-ups showing the actual visual designs for each screen. The primary researchers (VG and PW) were consulted at each iteration for feedback. All the requirements of the prototype were required to be met.

### Development of the Prototype

#### Deployment of an Open-Source Machine Learning Model for Image-to-Recipe Retrieval

An Apache-2.0 license is an open-source license, allowing users to freely use, modify, and distribute the products without restrictions [54]. Thus, a recently published cross-modal image-to-recipe retrieval model under an Apache 2.0 license [50] was deployed in this study. The deployed machine learning model is an end-to-end model based on hierarchical transformer-based encoders for images (eg, food photos) and text (eg, titles of recipes and ingredients). This model is composed of an image encoder, a recipe encoder, and the training objectives of supervised loss for paired data and self-supervised recipe loss. The results of the experiments demonstrated that the training objectives leveraging semantic relationships within recipes achieved 93% accuracy in the image-to-recipe retrieval task on the Recipe1M data set [50,55].

The primary requirement for deploying the machine learning model was to retrieve food items in the food photos. The psychometric properties (eg, validity and reliability) of image-to-recipe retrieval were not assessed in this study, as such evaluations were beyond the scope of this research. The code for the machine learning model was downloaded from a publicly available source [50]. Originally, the code was to generate the metrics for evaluating the performance of the model on the benchmark. We modified the code for the image-to-recipe retrieval model using PyTorch [56] on the Gadi Supercomputer at the National Computational Infrastructure of Australia. The output of the image-to-recipe retrieval model was a list of potential ingredients in a food photo.

The subset of Recipe1M+ with nutrition information [55] was used to perform the image-to-recipe retrieval in this study. The ingredients of 50,637 recipes in the subset were matched with the United States Department of Agriculture National Nutrient Database for Standard Reference release 27 [55]. The healthiness of the recipes was evaluated and presented using the traffic lights system established by the Food Standards Agency based on the content of saturated fat, sodium, sugar, and total fat per 100 g [55]. To assign Australian food composition data, the ingredients in the subset of Recipe1M+ with nutrition information were manually matched to a single ingredient or to a recipe listed in the Australian Food, Supplement and Nutrient Database (AUSNUT) 2011-13 database [57] based on the published method [58]. The Food and Agriculture Organization International Network of Food Data Systems Guidelines for Food Matching have been applied [59]. The matching guidelines provided criteria and a confidence code for each ingredient based on how well it matched with another food item. After removing the duplicates, a total of 352 food items in the subset of Recipe1M+ were matched with the AUSNUT 2011-13 database. More than three-quarters (270/352, 76.6%) of the food items were matched with exact food items. Approximately 7.4% (26/352)of the food items were matched with alternative food items, but 15.9% (56/352) of the food items were matched with similar foods.

#### Development Tools

Cloud computing provides on-demand access to internet infrastructure that enables users to access computing resources [60]. Given that cloud computing is required to run the machine learning model for image-to-recipe retrieval, a progressive web app (PWA) was developed. PWAs are multiplatform web apps published by the Google Web Framework [61]. It can operate on most mobile and desktop platforms (Android, iOS, Windows, and Linux) using a web browser [62]. In addition to the usual web app components, a PWA consists of a manifest file and service worker [61]. The manifest file assists in installing the app on the home screen of a device, providing faster access and a richer experience for users without requiring them to download it through an app store. A service worker is a JavaScript file that takes requests from the app. Thus, a PWA can provide users with an experience that is similar to that of a native mobile app.

The front-end languages, including HTML, cascading style sheets, JavaScript, and jQuery, were used to develop the prototype. The system used the MySQL database engine for data storage. The back end was developed using the Flask Python framework [63]. The Amazon Web Services (AWS) cloud (Amazon Web Services Inc, 2022) has become a popular and reliable platform for data storage, high-performance computing, and analytics [64-66]. The AWS Elastic Compute Cloud (EC2) has been used to run central processing unit–intensive cloud-based applications [67]. In this study, the AWS cloud services were used to host the back-end prototype.

### Evaluation of the Prototype

#### Ethics Approval

Ethics approval was granted by the University of Wollongong Human Research Ethics Committee (2022/327).

#### Participants

Participants were recruited through convenience sampling from the Illawarra community, a major coastal region 70 km south of Sydney, Australia. Social media from the University of Wollongong were used to advertise the study. The inclusion criteria were adults aged 18 to 65 years who own a smartphone and were willing to use the prototype and trial the prototype thoroughly. The exclusion criteria included inability to communicate in English, impaired ability to participate in the study, and having difficulties or major impediments to participating in the study’s components. APDs were also included in this study. All participants provided written informed consent to participate in the study. Each participant was given a deidentified participant code after completing the study.

#### Study Procedure

A semistructured, cross-sectional evaluation led by an experienced facilitator (VG) was conducted to explore participants’ opinions on the prototype in a face-to-face meeting or via the web-based meeting platform Zoom (Zoom Video Communications Inc, 2022) as chosen by the participants. The Mobile Application Rating Scale (MARS) offers a multidimensional measure of the quality of health-related apps [68]. This questionnaire can also be used as a checklist for the design and development of health-related apps [68]. The MARS has been applied to evaluate health-related apps with end users in many studies [69-72]. Thus, the MARS was used as a list of closed-ended questions to explore the participants’ opinions on prototype quality and the perceived impact of the prototype. The instructions for the questionnaire were followed to conduct this study using the participants’ smartphones. The prototype was accessed through a web browser. Subsequent semistructured in-depth interviews were conducted to deliberate and discuss the participants’ opinions on the design and perceived impact of the prototype. The interviews were audio recorded.

#### Data Analysis

A numerical summary of the responses to the MARS was created to provide a descriptive summary. Given that this study aimed to evaluate a prototype, the questions of accuracy of app description in the app store, goals, and evidence based on the informational content were excluded from the analysis. The semistructured interviews were transcribed verbatim. A process of coding and thematic analysis were performed to identify dominant themes [73]. The transcripts from adults and dietitians were pooled for joint coding. This method enables an in-depth understanding of user experience [19]. Exemplar quotes for the themes were identified and reported. All themes were managed and reviewed using the qualitative analysis software NVivo (version 12; QSR International Pty Ltd).

## Results

### Prototype Requirements

A summary of the final requirements is presented in Textbox 1. The primary requirement of Nutrition Assessment for the food layer of the NCP in the prototype was to transform the way that dietary data were collected, shifting from an active method of manually adding food items to a more passive approach of automatically retrieving food items from a food photo. The aim of this requirement was to reduce user burden of logging dietary intake. Meal patterns appear to be closely linked to eating habits [39]. Thus, food choice events were labeled as meals (eg, breakfast, lunch, dinner, or snacks). For Nutrition Diagnosis in the food layer of the NCP, a computational system was developed to facilitate continuous assessment of dietary intake and enable real-time feedback on food choices. This system used user input (dietary intake data) and logical rules (satisfaction rules) to determine compliance with personal food choice goals. For example, the system assessed whether a user had met their goal for a specific food group based on their reported dietary intake. This information was then used to provide real-time feedback on food choices. Both the internal-focused strategies (goal setting, self-monitoring, and rewards) and the external-focused strategies (environmental support and reinforcement) of CBT [37] were integrated into the design of the Nutrition Intervention. Given that the use of persuasion strategies and techniques is determined by the goals of information systems [22], the design principles of primary task support, dialogue support, and system credibility in the PSD model [22] were applied in this study. For example, the app was used as a tool to persuade users to change their eating behavior by making it easier to track their food choices. The prototype encouraged human-app interaction to help users keep moving toward their food choice goals by using gamification principles.

**Food layer of the Nutrition Care Process and requirements for the prototype.**

**Nutrition Assessment or Reassessment**

- Log daily foods and beverages organized by meals
- Take and upload food photos to retrieve food items in the food photos
- Log dietary intake manually
- Store and display the recently used food items to offer adaptive diet history records

**Nutrition Diagnosis**

- Automatically calculate daily intake values of food choices (food choices included 5 food groups [vegetables, fruits, grains, meat and alternatives, and milk and alternatives] and discretionary foods listed in the Australian Dietary Guidelines) based on dietary intake input
- Automatically compare daily dietary intake to personal goals for food choices

**Nutrition Intervention**

- Provide personalized information on the Australian Dietary Guidelines recommended number of daily servings for food choices based on age and gender
- Set personal goals for numbers of daily servings for food choices
- Provide immediate feedback to visualize the progress of daily dietary intake against personal goals of food choices
- Provide rewards when personal goals met
- Display information on the Australian Dietary Guidelines
- Search and display recipes

**Nutrition Monitoring or Evaluation**

- Provide 24/7 access
- Set up an account to allow user tracking of food choices on a personal basis
- Store and display daily intake over time to allow users to view their food choice history
- Provide challenges to affirm the user’s commitment to use the prototype

### Development of the Prototype

#### Structure of the Prototype

**Figure 1:**
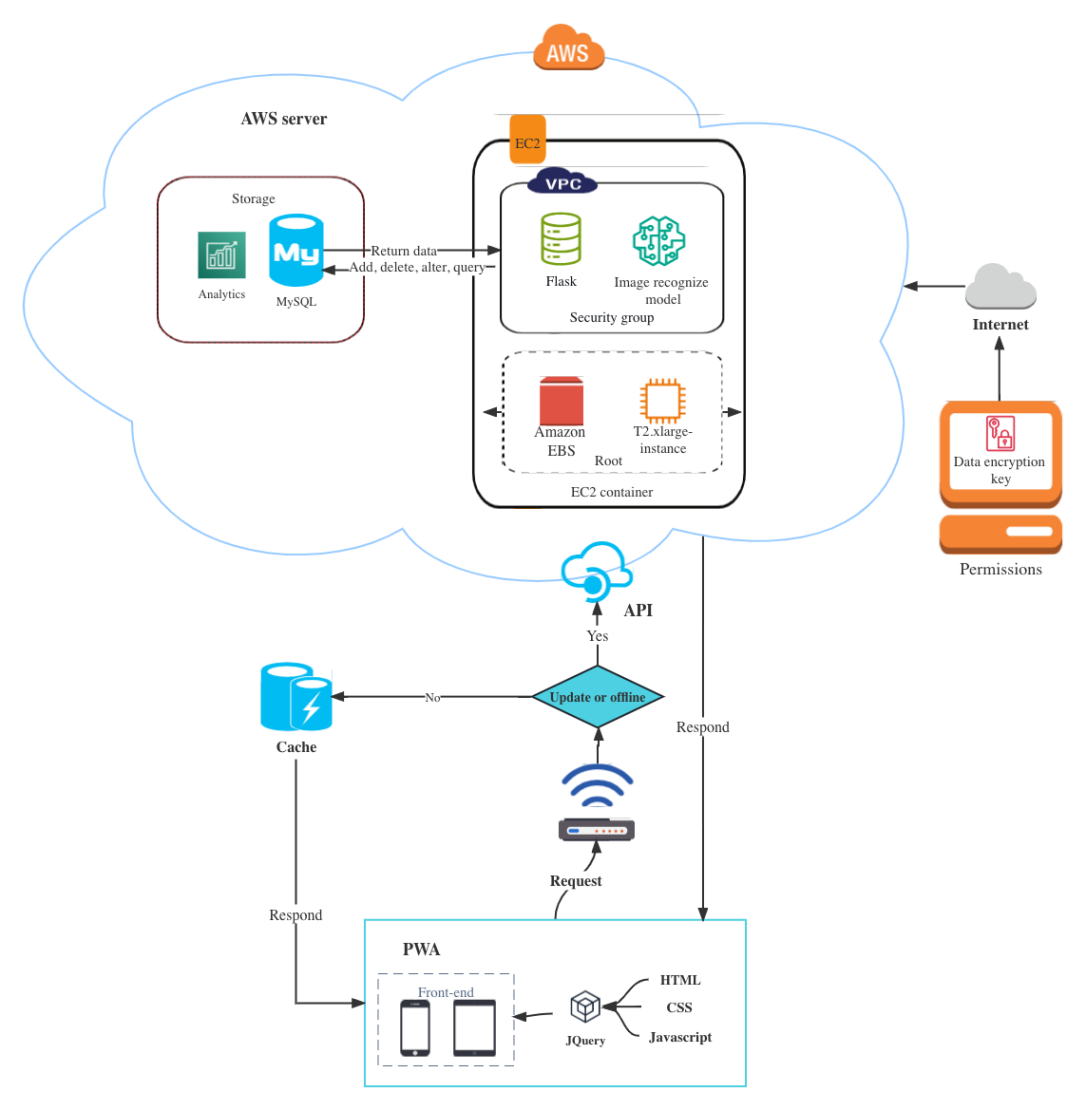
Schematic structure of the prototype. API: application programming interface; AWS: Amazon Web Services; CSS: cascading style sheets; EBS: Elastic Block Store; EC2: Amazon Elastic Compute Cloud; PWA: progressive web app; SQL: Structured Query Language; VPC: virtual private cloud.

The structure of the prototype is illustrated in Figure 1. The prototype was developed as a web application. The prototype became a PWA when a manifest file and service worker were used. Users can create an app icon on the home screen of their smartphone. Although the prototype can be downloaded and immediately run offline as an app, it required computing resources for running the image-to-recipe retrieval. The primary requirement of a manifest file was to create an app icon on the home screen, serving as a shortcut of the prototype. The prototype was further enhanced using the cache facilitated by the service worker. For example, the site displayed the content in a weak or offline network.

A virtual server in the cloud was built using EC2 in the Asia Pacific Sydney region. We chose a generic T2.xlarge on-demand instance with 4 central processing unit cores and 16 GB memory. CentOS 7 (Linux) was used as the operating system in the EC2 instance. An Amazon Elastic Block Store of the gp2 type was used to provide persistent block storage volume for the EC2 instance. The Flask framework was used as the back-end framework and was connected to the machine learning model of the image-to-recipe retrieval. An EC2 Key Pair was used to securely authenticate a user to the instance using the Secure Shell protocol. The EC2 Security Groups recommended by AWS were used as a virtual firewall to control inbound and outbound traffic to the instance. An EC2 Load Balancer was used to distribute incoming traffic to improve the availability and fault tolerance of the prototype. Asynchronous JavaScript techniques were used across the system to facilitate asynchronous data transfer between users and the server. The code (HTML, cascading style sheets, and JavaScript) was stored in the cloud. The food composition databases [57,74,75] and the subset of Recipe1M+ with nutrition information [55] were stored in the MySQL database. The data (input and output) were stored in a MySQL database.

#### Design of the Prototype

**Figure 2:**
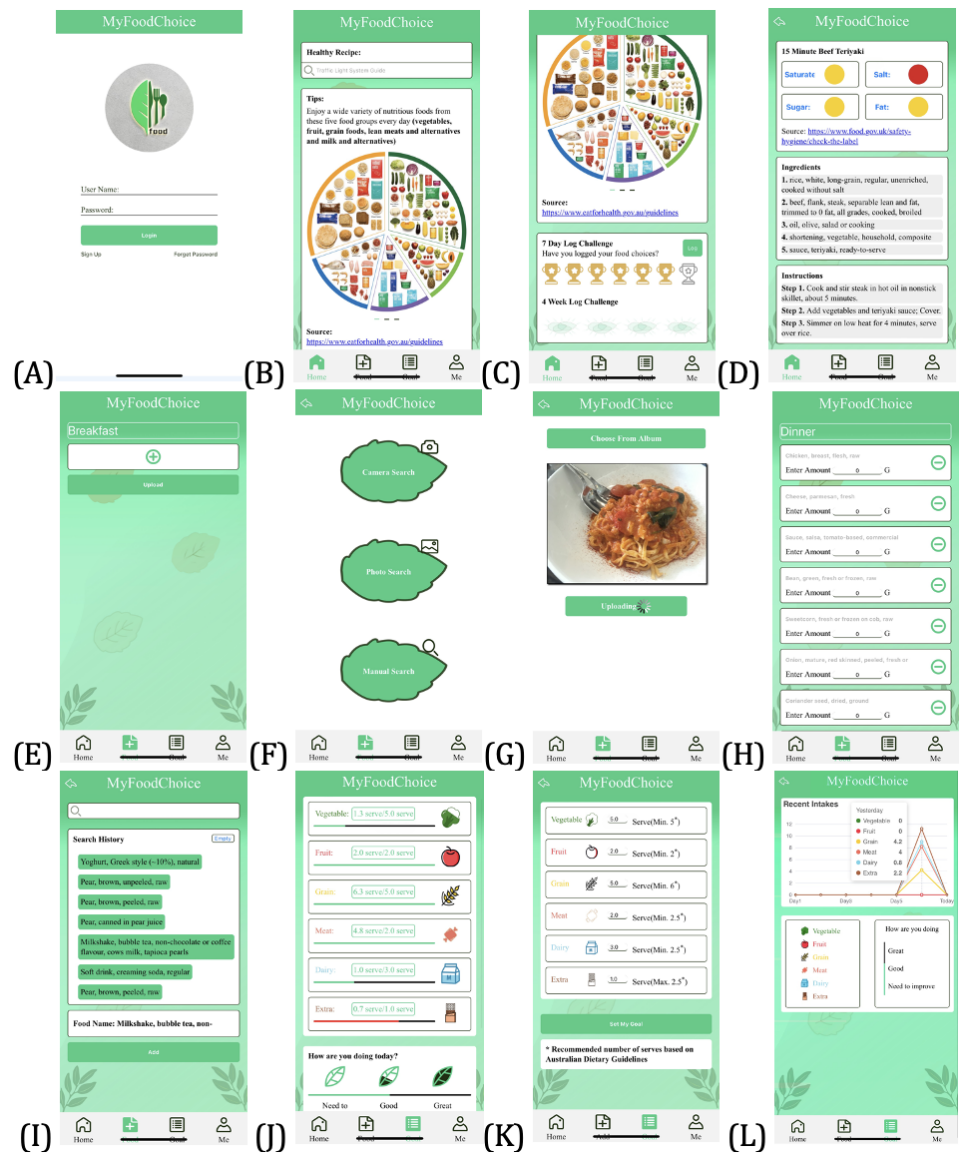
A sample of screenshots from the prototype. (A) Log-in screen; (B) home screen displaying recipe search and tips; (C) home screen displaying challenges; (D) example of recipe search output; (E) meal logging; (F) 3 methods for logging food intake; (G) example of uploading a photo for image-to-recipe retrieval; (H) example of image-to-recipe retrieval output; (I) example of storing history of food search; (J) feedback screen of daily intake of food choices; (K) goal setting screen; (L) feedback screen of weekly food choices.

**Figure 3:**
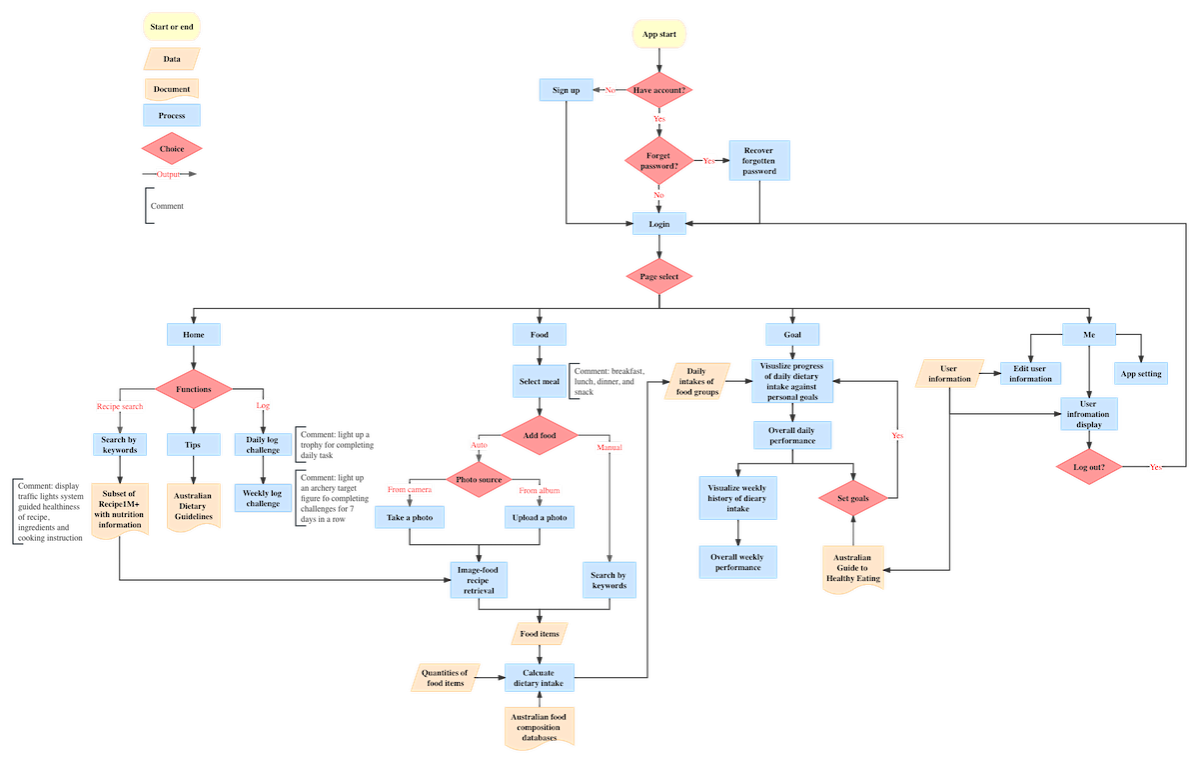
Information architecture of the prototype.

Screenshots of the screens are presented in Figure 2. The details of the information architecture are shown in Figure 3. On the home screen, to provide environmental support for food choices, users can search and select recipes to obtain recipe suggestions (Figure 2B). The suggestion included information on the healthiness of the recipe, an ingredient list, and cooking instructions (Figure 2D). The subset of Recipe1M+ with nutrition information [55] was used to provide recipe suggestions. The ADG, Guideline 1, Guideline 2, and Guideline 3 [3] were displayed as “Tips” on the home screen to reinforce the recommendations of the ADG (Figure 2B). The Australian Guide to Healthy Eating poster [76] was used to present relevant information related to the guidelines. The source of the recommendations was displayed on the screen. A 7-day challenge was created to ask users to actively log in the challenges (Figure 2C). Trophies lighted up for daily challenges as rewards. A 4-week challenge was included to display a historical record of the completed challenges. An archery target figure appeared for logging into the challenges for 1 week (7 days).

To log dietary intake, food intake was recorded as user-defined meals including breakfast, lunch, dinner, and snacks (Figure 2E). Users input their food items by taking a real-time food photo, uploading a food photo stored on the smartphone, and selecting a food item from a drop-down food list based on the food names from the AUSNUT 2011-13 database [57] (Figure 2F and 2G). The users were allowed to use these methods simultaneously. If the image-to-recipe retrieval function was used (eg, taking or uploading a food photo), users edited the retrieved food items from the food photos (Figure 2H). Then, users input the quantities of their food consumption. The prototype stored and displayed the recently used food items to offer adaptive diet history records (Figure 2I), which made food logging quick and simple.

The analysis of dietary intake was automatically performed. The Australian Health Survey–ADG food composition database [75] was applied to aggregate dietary intake data for 5 food groups (vegetables, fruits, grains, milk and milk alternatives, and meat and meat alternatives). Intakes of discretionary foods were identified using the Discretionary Food List database [74] and AUSNUT 2011-13 database [57]. One serving of discretionary foods is defined as containing 600 kJ or 10 g of alcohol, respectively [3]. The prototype stored and displayed the daily dietary intake over 7 days to allow users to view and self-monitor the history of their food choices (Figure 2L).

In goal setting, the prototype provided information on the ADG recommended number of daily servings for 5 food groups and discretionary foods based on the user’s age and gender (Figure 2K) [77,78]. The source of the recommendations was displayed on the screen. Users set their personal goals for 5 food groups and discretionary foods. The progress bar charts were used to provide immediate feedback to visualize the progress of dietary intake against the personal goals of each food group (Figure 2J). The icons of the 5 food groups and discretionary foods were initially displayed as whited-out icons (Figure 2J). If the logged dietary intake met personal goals, then the icons were changed to colored icons. The prototype also displayed daily and weekly overall performance in achieving personal goals. The parameters were as follows: (1) if dietary intake met the daily or 7-day average of 2 goals, then it displayed the “need to improve”; (2) if the dietary intake met the daily or 7-day average of 4 goals, then it displayed “good”; and (3) if the dietary intake met the daily or 7-day average of all 6 goals, then it displayed “great.” Examples of the design related to the applied design principles of the PSD model [22] and the general gamification principles in health care [79] are presented in Tables 1 and 2, respectively.

**Table 1:** Examples of the prototype mapped to the design principles proposed in the Persuasive Systems Design model.

Design principle and definition [22]				Example
**Primary task support**				
	**Reduction**			
		System should reduce effort that users perform their target behavior.	Log and track food intake via taking and uploading food photos Automatically compare daily dietary intake to personal goals and provide immediate feedback on dietary intake	
	**Tunneling**			
		System should guide users in the attitude change process by providing means for action that brings them closer to the target behavior.	Provide feedback of the progress of daily dietary intake against personal goals	
	**Personalization**			
		System should offer personalized content and services for its users.	Provide personalized information on the recommended number of daily servings for food choices based on age and gender Ask users to set their own goals for food choices	
	**Self-monitoring**			
		System should provide means for users to track their performance or status	Store and display daily dietary intake over 7 days to allow users to monitor and view their dietary intake	
	**Rehearsal**			
		System should provide means for rehearsing a target behavior	Repeatedly track food choices and visualize the progress of daily dietary intake against personal goals	
**Dialogue support**				
	**Rewards**			
		System should provide virtual rewards for users to give credit for performing the target behavior	Give users a virtual trophy if they log in the challenge Change whited-out icons of food choices to colored icons, if dietary intake met personal goals	
	**Liking**			
		System should have a look and feel that appeals to its users.	Use picture icons of food choices Using the traffic lights system to guide the healthiness of recipes	
**System credibility support**				
	**Trustworthiness**			
		System should provide information that is truthful, fair, and unbiased.	Provide information based on the Australian Dietary Guidelines	
	**Expertise**			
		System should provide information showing knowledge, experience, and competence.	Display the sources of information	
	**Surface credibility**			
		System should have competent look and feel.	Contain a limited number of screens based on the food layer of the Nutrition Care Process	
	**Verifiability**			
		System should provide means to verify the accuracy of site content via outside sources.	Display the links of the sources of information	

**Table 2:** Examples of the prototype mapped to the elements of gamification in health care.

Gamification principle	Description [79]	Example
Adaptive game mechanics	System should adapt to a user’s performance.	Store and display recently used food items to offer adaptive diet history records
Appointment dynamics	System should ask users to finish a task in a certain amount of time.	Log food intake within 24 hours
Badges or medals	System should reward users with badges or medals for designed tasks.	Give users a virtual trophy if they log in the challenge
Immediate feedback	System should provide immediate feedback after completing designed tasks.	Provide immediate feedback to dietary intake against personal goals of food choices
Progress status	System should allow users to check their progress.	Use progress bar charts to visualize progress of dietary intake against personal goals for food choices Change whited-out icons of food choices to their colored icons, if dietary intake met the personal goals

### Evaluation of the Prototype

#### Study Participants

A total of 12 adults and 3 APDs completed the evaluations (November 2022 to December 2022). The adults consisted of 4 male individuals and 8 female individuals aged 20 to 40 years, with a median age of 25.5 (IQR 21-33.5) years. There were 7 adults who completed tertiary education. The dietitians were female, aged 46 to 49 years, and had worked as dietitians for a mean of 20 years. The length of the evaluations ranged from 23 to 44 minutes, with a median of 35 (IQR 30-40) minutes.

#### Survey Results

**Figure 4:**
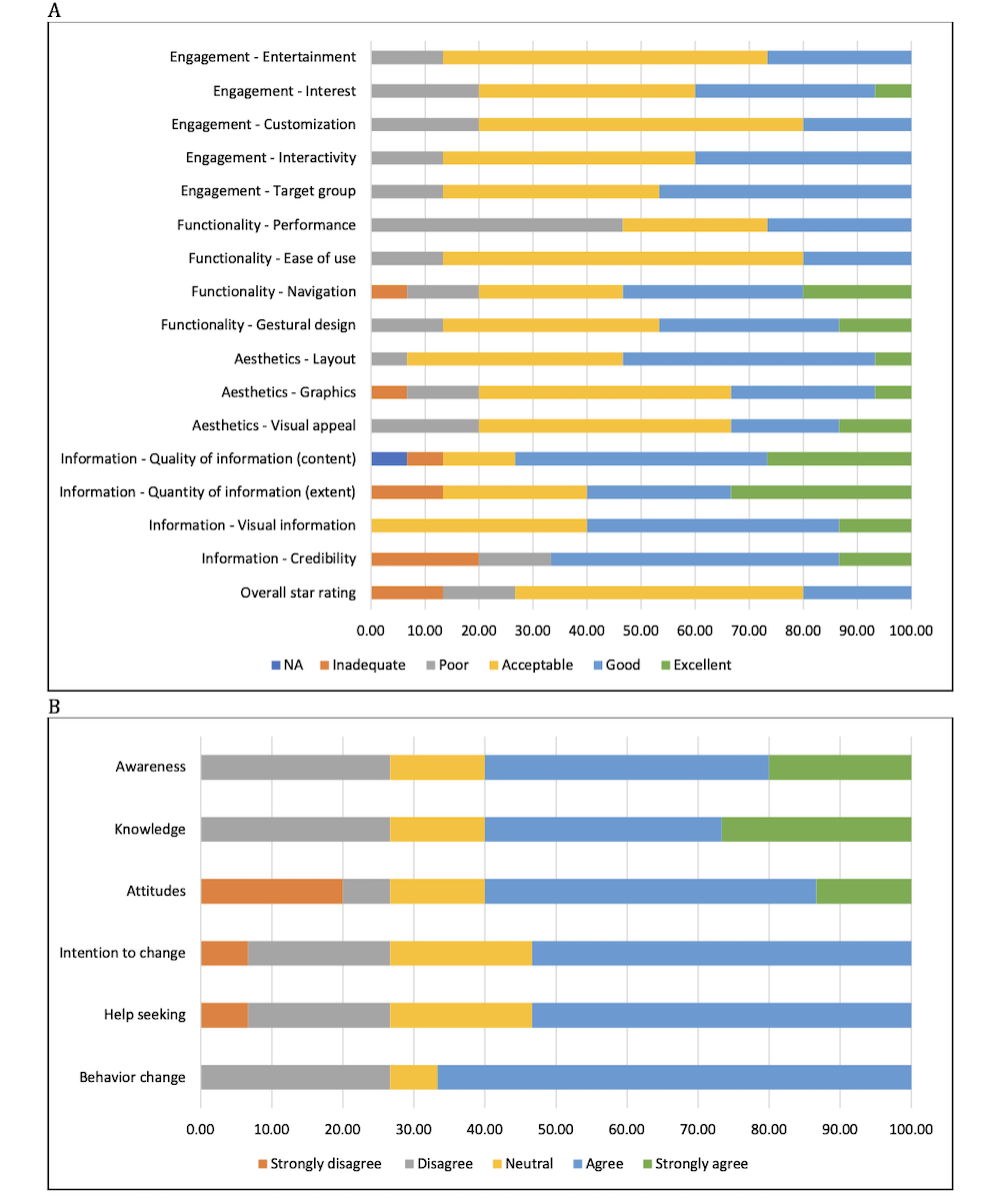
Participants’ statements regarding the prototype according to the Mobile Application Rating Scale (n=15). (A) Engagement, functionality, aesthetics, information, and overall star rating of the prototype; (B) perceived impact of the prototype related to healthy eating based on the Australian Dietary Guidelines.

The summary of the MARS scores for the prototype is presented in Table 3. The overall quality score was “acceptable,” with a median of 3.46 (IQR 2.78-3.81) out of 5 points. There were 3 participants who rated the prototype with 4 out of 5 stars (Figure 4A). For functionality, out of 15 participants, approximately half of the participants rated the navigation (n=8, 53%) and gestural design (n=7, 47%) of the prototype as good or excellent. However, the performance of the prototype showed potential for improvement, as 7 (47%) participants rated it poor. A total of 8 (53%) participants rated the layout of the prototype as good or excellent. Moreover, 60% (n=9) of participants rated the informational content of the prototype as good or excellent. A discrepancy was observed between the adults’ and dietitians’ opinions on credibility of the prototype. The median score for the perceived impact on healthy eating based on the ADG was 3.83 (IQR 2.75-4.08) out of 5 points. The feedback regarding the perceived impact of the prototype with respect to healthy eating based on the ADG is presented in Figure 4B. Both adults and dietitians advised that the prototype had the potential to impact awareness and knowledge of healthy eating based on the ADG.

**Table 3:** Summary of the Mobile Application Rating Scale scores applied to the prototype.

			Adults (n=12), median (IQR)		Dietitians (n=3), mean (SD)		Total (N=15), median (IQR)
**Engagement**							
	Entertainment	3 (3-3)		3.67 (0.58)		3 (3-3.5)	
	Interest	3 (3-4)		3.33 (1.15)		3 (3-4)	
	Customization	3 (3-3.25)		2.67 (0.58)		3 (3-3)	
	Interactivity	3 (3-4)		3.00 (1.00)		3 (3-4)	
	Target group	3.5 (3-4)		3.00 (1.00)		3 (3-4)	
	Mean score	3.1 (2.95-3.65)		3.13 (0.83)		3.2 (2.9-3.7)	
**Functionality**							
	Performance	2.5 (2-4)		2.67 (0.58)		3 (2-3.5)	
	Ease of use	3 (3-3.25)		2.67 (0.58)		3 (3-3)	
	Navigation	4 (2.75-4.25)		3.33 (0.58)		4 (3-4)	
	Gestural design	3.5 (3-4)		3.33 (0.58)		3 (3-4)	
	Mean score	3.5 (2.69-3.81)		3.00 (0.50)		3.5 (2.63-3.63)	
**Aesthetics**							
	Layout	4 (3-4)		3.00 (0)		4 (3-4)	
	Graphics	3 (3-4)		3.00 (1.00)		3 (3-4)	
	Visual appeal	3 (2.75-4)		3.33 (0.58)		3 (3-4)	
	Mean score	3.33 (2.92-3.83)		3.11 (0.51)		3.33 (2.83-3.67)	
**Information**							
	Quality of information (content)	4 (3.75-4.25)		3.33 (2.08)		4 (3.5-4.5)	
	Quantity of information (extent)	4 (3-5)		3.00 (2.00)		4 (3-5)	
	Visual information	4 (3-4)		3.67 (0.58)		4 (3-4)	
	Credibility	4 (3.5-4)		2.33 (1.53)		4 (2-4)	
	Mean score	4 (3.13-4.31)		3.08 (1.51)		4 (3-4.38)	
Prototype quality mean score			3.48 (2.8-3.82)		3.08 (0.83)		3.46 (2.78-3.81)
Overall star rating			3 (2.75-3)		3.00 (1.00)		3 (2.5-3)
**Perceived impact of the prototype**							
	Awareness	4 (2.75-4)		4.00 (1.73)		4 (2.5-4)	
	Knowledge	4 (2.75-4)		4.00 (1.73)		4 (2.5-4.5)	
	Attitudes	4 (2.75-4)		2.67 (1.53)		4 (2.5-4)	
	Intention to change	4 (2.75-4)		3.00 (1.00)		4 (2.5-4)	
	Help seeking	4 (2.75-4)		3.00 (1.00)		4 (2.5-4)	
	Behavior change	4 (2.75-4)		3.33 (1.15)		4 (2.5-4)	
	Mean score	3.75 (3.13-4.04)		3.33 (1.30)		3.83 (2.75-4.08)	

#### Interview Results

In-depth interviews lasted between 4.50 and 22 minutes, with a median of 7.75 (IQR 7.15-10.75) minutes. The participants elaborated on their experience of using the prototype, with a focus on prototype design.

##### User Experience of Using the Prototype

The results demonstrated that the prototype design evoked both positive and negative user experiences. For example, the participants found using the prototype fun and enjoyable:

It was overall really fun to use and trial.

Adult 10

I really did enjoy it.

Adult 11

The main driver of the positive user experience was interaction design, which provided immediate feedback on the progress of daily dietary intake against personal goals. This interaction design integrated the aesthetic elements into the gamification, creating an entertaining, interactive, and visually appealing experience of tracking food choices:

It has like the colored bars that tell you how far along you are reaching that the serve goals. So, it’s kind of like a visual. You can say, oh, I’m this close to getting it. I really liked that.

Adult 5

The participants found that the provision of recipe ideas was helpful. Suggested improvements included a need for a shopping list, images of the recipes, and a demonstration video; an option to upload, review, and rate recipes; and suggestions for quick 10-minute recipes:

I really liked when you did go into a recipe, it said, had like a green yellow system, like a traffic lights system. And then, yeah, it was just really simple recipe that seemed which was really helpful.

Adult 1

The analysis revealed that confusion when using the prototype was the major contributor to the negative user experience, which hindered the participants’ ability to learn how to use the prototype. The participants suggested including embedded instructions to provide guidance on how to use the prototype. Furthermore, the findings suggest that the current navigation design caused this confusion. The poor navigation design affected the performance of the prototype:

It did take a while for me to sort of work out what to do. So maybe if there were some more instructions first up, like if sometimes you go into an app and it sort of you get these navigation comments, go here, go here, go here ... may be a prompt to say this is where you go to.

Dietitian 1

I ticked some functions work that lagging or contains major technical problems [in the questionnaire]. I don’t think that’s true. They’re not lagging and it doesn’t contain major technical problems. It just takes a little bit of time for me to figure it out. So that is slowing my performance down. It makes it more time consuming to enter.

Dietitian 3

The feedback on the gestural design of the prototype showed that the participants had high expectations for the functions embedded in the touch screen of a smartphone. For example, the participants scrolled and stretched the images for information:

I couldn’t really see the pictures [on the homepage], like to zoom in to see what it was saying.

Adult 1

Furthermore, the participants were excited about the innovation of image-based dietary assessment in the prototype and valued its potential to reduce user burden for logging dietary intake. However, the participants were concerned about the validity and reliability of the image-based dietary assessment and the slow loading time of the food photos:

I think that’s, that’s [image-based dietary assessment] new. I haven’t seen that before. I think my concern is like kind of, detect ethnic meals, like say if someone would have like a traditional Asian dish, everything mixed together. I’m just wondering, can that detect as compared to like a western dish where everything’s nicely portioned out.

Adult 4

I couldn't upload the photo because too big ... If the one [image-based dietary assessment] is working, yeah, I would like it very much. Because I hate entering it [dietary intake] manually.

Adult 6

##### Perceived Impact of the Prototype on Healthy Eating

Almost all the participants reported that the prototype was useful for increasing their knowledge and awareness of healthy eating based on the ADG:

Definitely tracking your food and understanding the [Australian Dietary] guidelines.

Adult 3

I think the biggest one would be education about what the dietary guideline is and understanding what food groups are and giving them a reality check of how many of each group was that need to be consuming a day.

Dietitian 2

The perceived impacts on healthy eating based on the ADG were reported to depend on the use of a gain-framed approach, with a focus on food choices based on the ADG and tracking food choices with immediate feedback:

It shows you how much each of the core food groups that you’ve eaten each day. I think that will be a really useful tool for people. Really simple. And, yeah, that feedback would be really good ... I think you know, there are other apps that are available, but are probably a bit more complicated, and they concentrate on kilojoules ... taking the emphasis back to whole foods and with the push now for, you know, less ultra-processed foods. Yeah, I think there's a really good distinction there between.

Dietitian 1

However, the dietitians suggested that using the current version of the prototype was unlikely to change the attitude and eating behavior toward healthy eating based on the ADG. Suggested improvements included the provision of personalized tips on food choices and the use of reminders:

For example, if I just saw that I’d had 3.5 serves of meat. And in order to get under that, do I need to cut my meat intake in half or do I need to cut it by a third? So that kind of, in order to make behavior change happen, it’s not clear what I need to do. That’s probably the missing step. And maybe a tip that comes up to help them with that might be helpful. Or even just a motivational message, “great work, you’ve had your five vegetables today, here’s some other examples of servings of veggies,” for example.

Dietitian 3

For example, if a client, you know, I guess when they’re entering the target goals for food groups, for example, three serves of vegetables a day is their target, and then there is a day where they haven’t met it. And they have the app, and pop up for visual prompts for example, you only consumed, as a reminder, I guess, you know, you’ve only had one of your three target goals. One serve of the vegetables a day, and something like that. Something very simple, but it just reminds them that, oh, I haven’t had enough veggies today.

Dietitian 2

## Discussion

### Principal Findings

To our knowledge, this study was the first to describe the detailed development process and design of a PWA to provide real-time, personalized, and evidence-based support for self-managing food choices using persuasive technology, computer vision, and cloud computing. Combining theoretical frameworks with cross-disciplinary collaboration in the fields of nutrition and dietetics, computer science, and information technology have resulted in a novel, evidence-based prototype for personalized dietary advice. The elaborations on the user experience of using the prototype have provided an in-depth understanding of the roles of the design and its potential impact on healthy eating based on the ADG. A revised version of this prototype is currently under development.

The qualitative results suggest that the prototype can be used as a supportive tool to improve the awareness and knowledge of the ADG. The ADG were developed through systematic literature reviews, which follow transparent processes to identify, evaluate, and synthesize relevant available research on specific questions in diet-disease relationships [3]. In the ADG, food modeling was also applied to model the nutrient reference targets based on diet scenarios (eg, foundation diets, total diets, rice-based and pasta-style diets, and plant-based diets) using a linear programming and constraint optimization technique [80]. The quantitative recommendations bring together all food consumption patterns from national surveys, the recommended dietary intake values, and evidence related to the potential impact of food consumption on health [80]. Food consumption patterns from national surveys were used in the modeling to account for food supply and food preference. The outcomes of food modeling provided the foundation diets, including 5 food groups, further constructed to form total diets with discretionary foods. However, the food environment and food systems have changed rapidly in recent years, such as increased availability, accessibility, and affordability of energy-dense foods, and intense marketing of such foods has become common [81,82]. Thus, the strength of the ADG lies in their reliance on food modeling based on national food consumption survey data and the body of scientific evidence from published studies on health and disease prevention. This is partly a theoretical strength. In practice, there appears to be insufficient knowledge and consensus among adults in Australia on how to achieve a healthy diet [4,5]. Thus, a supportive tool is required to translate the ADG into daily practice. Incorporating the ADG into the prototype provides users with evidence-based dietary recommendations on food choices. This has improved the quality of the prototype in this study and offered a supportive tool that can work within the food system to provide personalized practical advice for food choices in real time for adherence to the ADG.

In this study, a prototype was successfully developed and integrated the food layer of the NCP, CBT strategies, and the ADG into the actual prototype features guided by the PSD model and a gain-framed approach. The goal of using apps for nutrition intervention tends to automate and advance current evidence-based practice by leveraging the rapid development and maturation of digital tools [83,84]. The apps for nutrition interventions tend to provide ongoing support for behavior changes on a daily or on-demand basis by offering the features such as dietary tracking, goal setting, immediate feedback, and environmental support [9]. The results of the evaluations highlighted that the prototype simplified the process of goal setting and self-monitoring of personal food choices. The capabilities of tracking food choices in the prototype were further enhanced by integrating a state-of-the-art machine learning model for retrieving recipes from food photos. Although dietary intake was still self-reported, its objectivity may be improved owing to the presence of food photos [47]. Providing real-time feedback on food choices using apps can help users adopt healthy eating by enabling them to immediately observe the link between dietary intake and compliance with their personal goals. This approach appears to encourage healthy eating without coming across as preachy [21].

Moreover, the qualitative results suggest that the prototype provides a supportive environment for gaining an entertaining and interactive experience in healthy eating based on the ADG by leveraging gamification principles. Gamification in health care has become increasingly popular in recent years [79]. Gamified interventions tend to illustrate target behavior, simplify the intervention to manageable tasks, provide immediate feedback, and reinforce good performance [85]. Thus, gamification makes complex tasks or goals more engaging and enjoyable and has the potential to empower individuals to make positive behavior changes [86]. By incorporating the aesthetic design elements into the gamification elements of the prototype, users are more likely to be engaged and motivated. The emotional aspects of interaction design appear to assist in creating a stronger connection between users and the app and encourage users to achieve their goals. Adopting this approach assists in improving adherence to using an app over time.

Despite this, the qualitative findings of the evaluation revealed that high-level personalization is required to change attitudes and eating behavior toward healthy eating based on the ADG. In this study, users set their personal goals for food choices and track their progress of personal goals based on their dietary intake at meals. Thus, the dietary advice received by a user is personalized based on their dietary intake. However, the dietitians have suggested that high-level personalization (eg, specific food choice and its portion size) is required for eating behavior changes. With advances in machine learning, high-level personalized dietary advice can be provided by using collaborative filtering–based methods, content-based methods, context-awareness methods, and hybrid methods [48]. For example, the collaborative filtering–based method has been applied to model the interactions between a user and food items for recommendation [48]. The context-awareness approach has been used to explore the characteristics of dietary patterns by leveraging information on social media for food recommendations [48]. Future studies are required to understand users’ needs for high-level personalized dietary advice leveraging digital tools.

Furthermore, the results suggest that navigation was a crucial part of the overall experience of using the prototype. Navigation plays a critical role in learnability, discoverability, and user engagement when using a digital tool [87]. It provides a way for users to access the different features and functionality of the app and helps them find the information they need [88]. In this study, a discrepancy was observed between participants’ opinions on navigation in the MARS scores and in-depth interviews. This may be due to the limitations of the methods used to evaluate the prototype. Participants may not accurately reflect their true experiences in the survey. The present findings of the in-depth interviews tend to provide a deeper understanding of the user experience.

In this study, although users were not involved in developing the prototype, the prototype was developed using a user-centered design approach. By adopting the user-centered design principle, users become the focus of the design process, and their perspectives are reflected in each step of development [89,90]. The requirements of this prototype were developed based on the lead dietitian’s previous work [39,44-46,52], which informed the design of the features of this prototype. This work has offered novel insights into users’ needs for dietary advice on food choices, which is beyond the traditional methods of gathering information through surveys and interviews with users at the initial stage of development. The previous work has added new dimensions to understand users’ needs for dietary advice on food choices. Involving users in the later stages of prototype development also allowed the team to focus on technology feasibility and rapid prototyping, resulting in a more efficient development process. The qualitative results of the evaluation suggest that the prototype was made available to the participants for evaluation, enabling them to directly engage with it and gain practical experience. This facilitated an in-depth understanding of the user experience and improved communication of users’ needs, thus enhancing the prototype in future iterations. Therefore, involving users in the later stages of prototype development assists in creating a digital health solution that balances users’ needs, requirements, and technical capabilities. The prototype can be a useful tool for both users and health care professionals as it can be readily embedded in the relevant health care system.

This study provides a practical example of integrating a recent, advanced machine learning model for image-to-recipe retrieval into an app for dietary assessment. Computer vision is a rapidly evolving field in recent years. Developing a new state-of-the-art machine learning model for image-to-recipe retrieval is a challenging task, which requires a substantial amount of time, expertise, and resources. In this study, a recently developed machine learning model under an Apache 2.0 license was integrated into the prototype. It not only saves time and cost but also allows for rapid technology feasibility assessment. Furthermore, Graphics Processing Unit (GPU) acceleration is required to improve the performance of image-to-recipe retrieval using the current state-of-the-art model. A GPU is a specialized processing unit that is optimized for processing data in parallel, which is effective for handling large amounts of data simultaneously [91]. Advances in computer vision and natural language processing have enabled the development of more sophisticated models for image-to-recipe retrieval, which have shown promising results in improving the accuracy of retrieval and generate higher-quality results [50]. However, the implementation of more complex machine learning models can be computationally demanding. Both image processing and natural language processing are required to run the model for image-to-recipe retrieval in this study. Moreover, current smartphones are typically equipped with high-quality cameras to capture high-resolution images. Thus, GPUs may be helpful in speeding up the process to improve the overall performance of image-to-recipe retrieval to assess dietary intake from food photos.

The detailed description of the development process and design of the prototype in this study offer valuable insights into the rapid development of a novel evidence-based mobile app for personalized dietary advice on food choices. Existing studies on developing mobile apps for nutrition interventions tend to lack information on the design of information systems [17,18]. Thus, developing an information system solution still requires intuition, experience, and trial-and-error methods [32]. The development of a mobile app as an experiment in design science research offers comprehensive insights into the nature of the problem and possible solutions for a broad audience [51]. The prototype developed in this study was formalized as a representation of information system problems, solutions, and solution processes by following the guidelines of the design science paradigm (Table 4). This formalization helped clarify the knowledge generated by this study.

**Table 4:** An Illustration of applying the design science paradigm guidelines to the study.

Guideline	Definition [51]	Application
Guideline 2: Problem relevance	To create technological solutions that address critical and applicable business issues	The aim of using a mobile app for nutrition interventions is to assist in users to make healthy eating easy to do. This is a relevant problem because the adherence to the ADG^a^ is poor in Australian adults. However, adherence to the mHealth^b^ nutrition interventions in real-world settings is low. There is a need for a novel mobile app to promote healthy eating based on the ADG.
Guideline 5: Research rigor	To apply rigorous methods in both the construction and evaluation of the design artifact	A co-design approach was applied by a team of researchers in computer science and information technology as well as an Accredited Practising Dietitian, with the latter taking the lead. The requirements of the prototype were developed based on the preliminary research work on food choices and the flow of generating dietary intake data. The requirements were also shaped by the integration of the food layer of the Nutrition Care Process, the strategies of cognitive behavioral theory and the ADG, along with insights from the reviews on the characteristics of apps for nutrition interventions. A gain-framed approach and the Persuasive Systems Design model were used to guide the design of the prototype.
Guideline 6: Design as a search process	To use available means to achieve desired outcome while satisfying laws in the problem environment	The prominent features of the prototype included the use of a recent machine learning model of image-to-recipe retrieval, food choice tracking with immediate feedback leveraging gamification principles, personal goals setting for food choices, as well as the provision of recipe ideas and information on the ADG.
Guideline 1: Design as an artifact	To generate a viable artifact in the form of a construct, a model, a method, or an instantiation	A prototype mobile app was created in this study that can be tested and evaluated.
Guideline 3: Design evaluation	To establish the utility, quality, and efficacy of a design artifact by applying well-executed evaluation methods	The evaluation of the prototype was the triangulation of methods with a combination of quantitative and qualitative methods and analyses.
Guideline 4: Research contributions	To offer clear and verifiable contributions in the areas of the design artifact, design foundations, and design methodologies	The design science contributions of this study are the prototype mobile app as the design artifact and the evaluation results in its usability. These contributions advance the understanding of how best to integrate the food layer of the Nutrition Care Process, behavior change theory, the ADG, a gain-framed approach, the design principles of the Persuasive Systems Design model, gamification principles, a recent machine learning model under an Apache 2.0 license and cloud computing resource into a mobile app prototype for personalized dietary advice on food choices.
Guideline 7: Communication of research	To effectively communicate the research to both technology-oriented and management-oriented audience	This study documents the detailed development process and insights into its creation for both technical and health-related audiences. This work provides a valuable example of developing a novel, evidence-based mobile app for personalized dietary advice on food choices using recent advancements in computer vision.

^a^ADG: Australian Dietary Guidelines.

^b^mHealth: mobile health.

### Limitations

Participants in the evaluation were recruited via convenience sampling. The findings are not generalizable to the larger population but rather provide insights into the specific context of the study. The sample size of the evaluation was small. Although data saturation was not pursued in this study, previous research has demonstrated that this sample size is effective in identifying the majority of the usability issues [92,93]. In this study, a major strength of the evaluation was the triangulation of methods using a combination of quantitative and qualitative methods and analyses. The reported usability problems in this study also overlapped substantially across participants. Qualitative research involves collecting in-depth, open-ended data from a small sample of participants to gain a deeper understanding of their experiences, attitudes, and perceptions [94]. Thus, conducting in-depth interviews with end users to evaluate a prototype offered a deeper understanding of how users interact with the prototype, including how they use it, what features they find useful, and what challenges they encounter. This provides insights into how to improve the usability and functionality of the prototype to better meet the needs of its users. In addition, qualitative research helps to identify any barriers or challenges that users may face when using the prototype, such as technical issues and navigation difficulties. Understanding these barriers helps to make improvements to the design and functionality of the prototype to address these concerns and improve user experience. Moreover, investigator subjectivity may have influenced the design of the prototype. Although the use of theories tends to be sufficient to guide the design of the prototype, future iterations can benefit from more complex development processes. Evaluating the psychometric properties of image-to-recipe retrieval may be required to gain insights into the validity and reliability of the image-based dietary assessment used in the prototype.

### Conclusions

This study describes the detailed development process and design of the prototype to provide personalized, evidence-based support for self-managing food choices from the ADG in real time. Through cross-disciplinary collaboration, a recently developed machine learning model for the image-based dietary assessment was integrated into the prototype. The food layers of the NCP, CBT strategies, and the ADG were then translated into the actual prototype features guided by the PSD model and a gain-framed approach. Participants reported that their experience of using the prototype was enjoyable and engaging. Both the users and dietitians appreciated the novel features of the prototype, such as using image-based dietary assessment and gamification principles for tracking food intake. The detailed description enhances the transparency of the prototype and provides detailed insights into its creation. This study provides valuable insights into developing a novel, evidence-based app for personalized dietary advice on food choices using the recent advancements in computer vision.

## Abbreviations

ADG: Australian Dietary Guidelines
APD: Accredited Practising Dietitian
AUSNUT: Australian Food, Supplement and Nutrient Database
AWS: Amazon Web Services
CBT: cognitive behavioral theory
EC2: Elastic Compute Cloud
GPU: Graphics Processing Unit
MARS: Mobile Application Rating Scale
mHealth: mobile health
NCP: Nutrition Care Process
PSD: Persuasive Systems Design
PWA: progressive web app

## Data Availability

Data cannot be shared publicly because the study analyzed qualitative data, and the participants did not consent to have their full transcripts made publicly available. Data requests may be sent to the principal investigator of the project, VG, or the University of Wollongong and Illawarra Shoalhaven Local Health District Human Research Ethics Committee.
